# Cross-Dataset Generalization of Deep Learning for Retinal Vessel Segmentation in Fundus Images: External Evaluation from DRIVE to CHASE_DB1

**DOI:** 10.3390/bioengineering13101215

**Published:** 2026-10-19

**Authors:** Rashid Abbasi, Oyewale Ibrahim Abdulrasheed, Madiha Jabeen Abbasi, Md Belal Bin Heyat, Farhan Amin, Isabel de la Torre, Mirtha Silvana Garat de Marin, Eduardo Silva Alvarado

**Affiliations:** 1 College of Computer Science and Artificial Intelligent, Wenzhou University, Wenzhou 325035, China; rashidd.abbasi@gmail.com (R.A.); oyewaleibrahim2@gmail.com (O.I.A.); 2 School of Basic Medicine, Wenzhou Medical University, Wenzhou 325000, China; jabeenmadiha44@gmail.com; 3 CenBRAIN Neurotech Center of Excellence, School of Engineering, Westlake University, Hangzhou 310018, China; 4 Department of Computer Science and Engineering, Yeungnam University, Gyeongsan 38541, Republic of Korea; 5 eHealth and Telemedicine Group, University of Valladolid, 47011 Valladolid, Spain; 6 Department of Projects, Universidad Internacional Iberoamericana, Arecibo, PR 00613, USA; silvana.mari@unic.co.ao; 7 Department of Projects, Universidad Europea del Atlántico, Isabel Torres 21, 39011 Santander, Spain; eduardo.silv@uneatlantico.es

**Keywords:** retinal vessel segmentation, fundus imaging, deep learning, U-Net, ResNet34, external validation, domain shift, cross-dataset generalization, retinal imaging, artificial intelligence

## Abstract

Automated retinal vessel segmentation can support quantitative analysis of fundus photographs and facilitate measurement of vascular characteristics associated with ocular and systemic disease. Although deep-learning methods achieve high segmentation performance on benchmark datasets, their reliability may decrease when applied to images obtained under different acquisition conditions. This study evaluated the cross-dataset generalizability of a U-Net segmentation architecture incorporating an ImageNet-pretrained ResNet34 encoder. Sixteen images from the DRIVE dataset were used for model training and four were retained for in-domain validation. Images were resized to 256 × 256 pixels and processed using a consistent normalization pipeline, while spatial and photometric augmentation was applied during training. Without target-domain retraining or domain adaptation, the trained model was subsequently evaluated on the independent CHASE_DB1 dataset. Segmentation performance was assessed using the Dice coefficient, intersection over union (IoU), and sensitivity, together with qualitative examination of predicted vessel maps. On the held-out DRIVE images, the model achieved a Dice coefficient of 0.7263, IoU of 0.5703, and sensitivity of 0.7201. On CHASE_DB1, performance decreased to 0.6269, 0.4573, and 0.5502, respectively. The disproportionately larger reduction in sensitivity indicated an increased frequency of missed vessel pixels under cross-dataset evaluation. Qualitative examination showed greater fragmentation and omission of fine and peripheral vascular structures in CHASE_DB1 images. These findings demonstrate that within-dataset performance may overestimate the robustness of retinal vessel segmentation models when transferred to a different imaging domain. External evaluation should therefore form an important component of retinal imaging artificial intelligence research. Future work should investigate higher-resolution processing, vessel-width-specific evaluation, multi-domain training, domain-adaptation strategies, and larger multicentre datasets to improve model generalizability.

## 1. Introduction

Diabetic retinopathy (DR) is a chronic microvascular complication of diabetes characterized by progressive retinal abnormalities that can ultimately lead to visual impairment and irreversible vision loss. The retinal circulation is particularly valuable for clinical and biomedical investigation because retinal microvascular structures can be directly visualized non-invasively using fundus photography. Alterations in vascular morphology, including vessel calibre, density, tortuosity, branching characteristics, and vascular complexity, may provide information about retinal and systemic vascular status. Consequently, accurate extraction of the retinal vascular tree represents an important foundation for quantitative fundus image analysis and computer-assisted assessment of retinal disease. Retinal vessel segmentation aims to distinguish vascular pixels from the surrounding retinal background and thereby provide a structural representation from which vascular characteristics can be quantified. Reliable segmentation is important because errors in vessel delineation can propagate into subsequent measurements of vessel width, density, branching, tortuosity, and other morphological features. Manual delineation by trained observers can provide detailed vascular annotations but is labor-intensive, time-consuming, and susceptible to inter-observer variation, particularly in regions containing narrow, low-contrast, crossing, or peripheral vessels. These limitations have motivated extensive research into automated retinal vessel segmentation methods. Deep-learning approaches have become especially prominent because they can learn hierarchical vascular representations directly from annotated images and have generally reduced reliance on manually designed image features [1]. Among deep-learning architectures, U-Net and related encoder–decoder networks have become widely used for retinal vessel segmentation. Their architecture combines progressively learned semantic features with higher-resolution spatial information transmitted through skip connections, making the framework well suited to biomedical structures that require pixel-level localization. Numerous extensions have subsequently incorporated residual connections, attention mechanisms, multiscale representations, feature pyramids, and deep supervision to improve vascular segmentation. For example, spatial attention has been integrated into U-Net to improve feature refinement when relatively limited annotated retinal data are available. Multiscale and feature-pyramid approaches have similarly been developed to improve representation of vessels with markedly different diameters. More recent architectures continue to combine multiscale processing and attention mechanisms specifically to improve the representation of small vessels and complex vascular structures [2]. Despite these advances, segmentation of fine and low-contrast retinal vessels remains challenging. The retinal vascular tree contains structures ranging from major arteries and veins to progressively narrower peripheral vessels that may occupy only a small number of image pixels. These smaller structures generally exhibit weaker contrast against the surrounding retinal background and may be adversely affected by image resizing, convolution, pooling, and repeated feature-map downsampling. As a result, a segmentation model may accurately recover prominent vascular branches while producing incomplete, fragmented, or missing predictions for smaller vessels. Previous studies have therefore specifically modified network structures to improve small-vessel preservation, including densely connected U-Net variants and architectures designed to retain multiscale vascular information [3]. The continuing development of architectures targeting small-vessel representation indicates that fine-vessel segmentation remains an unresolved technical problem. A related issue is the substantial class imbalance inherent in retinal vessel segmentation. Background pixels greatly outnumber vessel pixels in most fundus photographs, while fine vessels constitute an even smaller fraction of the image. Consequently, overall pixel accuracy can remain high despite omission of clinically or morphologically relevant vascular structures. Metrics that directly evaluate the vessel class, such as the Dice coefficient, intersection over union (IoU), and sensitivity, are therefore important for characterizing segmentation performance. In particular, sensitivity measures the proportion of reference vessel pixels correctly identified and can reveal increased false-negative segmentation under difficult imaging conditions. However, overall sensitivity should not be interpreted as a direct measurement of thin-vessel recall unless vessels are explicitly separated according to vessel width. Qualitative examination of segmentation maps can therefore provide complementary information about whether errors preferentially occur in peripheral, low-contrast, or fragmented vascular structures. Beyond fine-vessel segmentation, a major challenge for retinal imaging artificial intelligence is generalization across image domains. High performance on a benchmark dataset does not necessarily guarantee equivalent performance when a model is applied to images obtained using a different acquisition device, population, resolution, field of view, illumination environment, or clinical setting. Fundus datasets may differ substantially in image dimensions, colour distribution, retinal pigmentation, contrast, camera characteristics, annotation procedures, and participant populations. Deep neural networks trained using relatively small datasets may consequently learn visual characteristics that are specific to the development dataset rather than fully domain-invariant representations of retinal vessels. Cross-dataset generalization has therefore become an important consideration in retinal vessel segmentation research. This distinction is particularly important when interpreting performance reported on commonly used retinal datasets such as DRIVE, STARE, and CHASE_DB1. Evaluating a model separately on several datasets after training or fine-tuning it within each dataset demonstrates performance under dataset-specific training conditions, but it does not necessarily establish that the model can generalize to a genuinely unseen imaging domain. A more demanding experiment is to train the model using one source dataset and directly evaluate it on another dataset without target-domain retraining. Such evaluation provides a clearer estimate of performance degradation resulting from distribution shift. Previous research has demonstrated that preprocessing strategies, architecture design, and domain-generalization methods can affect the extent to which retinal vessel models retain performance across datasets. Knowledge-enhanced and multi-dataset approaches have likewise been investigated specifically to improve robustness across heterogeneous retinal datasets. Transfer learning provides another useful strategy when annotated retinal data are limited. Rather than training an encoder entirely from random initialization, representations learned from larger image datasets can be transferred to medical image segmentation tasks and subsequently fine-tuned. Pretrained convolutional encoders provide low-level representations of edges, textures, and shapes that can be adapted to retinal structures. Studies evaluating pretrained encoders within U-Net-type architectures have shown that transfer learning can improve segmentation performance when labelled retinal datasets are comparatively small [4]. Residual encoders are also attractive because residual connections facilitate optimization of deeper networks while enabling hierarchical feature extraction. However, a more powerful pretrained encoder cannot by itself guarantee robustness to differences between the source and target imaging domains. Image resolution represents an additional consideration in cross-dataset retinal vessel segmentation. Standardizing fundus photographs to a common input size simplifies model development and reduces computational requirements, but aggressive downsampling may remove or weaken small vascular structures. This issue becomes especially important when datasets with different native resolutions are transformed to the same low-resolution model input. Information loss during resizing may disproportionately affect narrow vessels that are already represented by only a few pixels. Thus, an apparent cross-dataset decline may reflect a combination of acquisition-domain differences, preprocessing effects, model representation limitations, and variation in vascular appearance. Evaluating the resulting errors is therefore useful not only for benchmarking model performance but also for identifying priorities for future model development. The present study addresses these issues by emphasizing evaluation of robustness rather than architectural novelty. A U-Net segmentation network incorporating an ImageNet-pretrained ResNet34 encoder was developed using the DRIVE dataset and subsequently evaluated on the independent CHASE_DB1 dataset without target-domain retraining or domain adaptation. The purpose was to determine whether segmentation performance obtained on held-out images from the development dataset was maintained when the same trained model was transferred directly to a different retinal imaging dataset. The same principal preprocessing and inference framework was retained across both datasets to reduce target-domain-specific optimization. Recent advances in retinal vessel segmentation have introduced architectural improvements beyond conventional convolutional encoder–decoder networks. Attention-based approaches, such as SA-UNet, incorporate spatial attention mechanisms to emphasize informative vascular regions and improve feature representation. Similarly, multiscale architectures aim to capture retinal vessels of different widths and morphological characteristics, which is particularly important for detecting thin capillaries and preserving vessel continuity. Hybrid convolutional neural network (CNN) and Transformer architectures have also emerged as promising alternatives. By combining convolutional feature extraction with attention-based global context modeling, these networks seek to capture both fine local vessel structures and long-range relationships across retinal images. However, such architectures may introduce additional computational complexity and can require careful optimization when only limited annotated training images are available. Another important research direction involves lightweight segmentation networks, which seek to maintain competitive vessel segmentation performance while reducing model parameters and computational requirements. Such approaches may be especially relevant for practical applications involving limited hardware resources. Although these architectural developments offer potential advantages, the present study adopts a U-Net model with an ImageNet-pretrained ResNet34 encoder as an established reference framework. The objective is not to introduce a new network architecture but to examine how a source-trained segmentation model performs when applied to an independent retinal image dataset without target-domain adaptation. Evaluating advanced attention-based, multiscale, hybrid Transformer, and lightweight architectures under the same external evaluation protocol represents a valuable direction for future work.

### Research Contribution and Novelty

The contribution of this study is primarily experimental and evaluative rather than architectural. Instead of introducing a new segmentation network, we investigate the cross-dataset generalization behavior of an established U-Net–ResNet34 framework trained on a limited DRIVE source dataset and evaluated independently on CHASE_DB1 without target-domain fine-tuning or adaptation. The study provides a focused assessment of segmentation performance under this transfer setting using Dice coefficient, IoU, sensitivity, and qualitative visualization. By examining the difference between internal validation and independent external evaluation, the work highlights the challenges associated with applying source-trained retinal vessel segmentation models to images acquired under different conditions. The findings provide a reference point for future investigations of more advanced architectures under comparable cross-dataset evaluation protocols.

Rather than interpreting qualitative observations as a formal measurement of thin-vessel recall, the analysis is used to identify potential failure mechanisms requiring more specific investigation. By examining how an established segmentation architecture behaves under a realistic cross-dataset shift, this work seeks to contribute to the broader evaluation of robustness in retinal imaging artificial intelligence. Demonstrating a performance decline on an independent dataset is not, by itself, evidence that the architecture is unsuitable for retinal vessel segmentation; rather, it indicates that strong within-dataset performance should not automatically be interpreted as evidence of reliable transferability. The results provide a baseline for future investigations involving higher-resolution processing, vessel-width-specific evaluation, domain adaptation, multi-domain training, more diverse retinal datasets, and larger multicentre image collections. Such work may ultimately help establish retinal vessel segmentation systems that are more robust to the heterogeneous imaging conditions encountered beyond their original development datasets.

The rest of this paper is summarized as follows. In Section 2, we survey the latest literature. Section 3 provides a detailed description of the proposed model. We present a performance analysis and discussion in Section 4 and a conclusion in Section 5.

## 2. Related Work

Recent advances in deep learning have substantially improved automated retinal vessel segmentation, particularly through encoder–decoder architectures based on [5], reviewed deep learning-based retinal vessel segmentation approaches and highlighted the importance of network architecture, feature representation, and the challenges associated with accurate detection of complex vascular structures. Ref. [6] introduced Spatial Attention U-Net (SA-UNet), which incorporates spatial attention and structured dropout to improve feature refinement and reduce overfitting when annotated training data are limited. Similarly, ref. [7] proposed a multiscale U-Net with spatial positional attention to improve vessel boundary identification and the extraction of vascular features at different scales. These studies demonstrate the effectiveness of attention-based and multiscale feature learning for retinal vessel segmentation.

More recent research has investigated architectural efficiency and the preservation of fine vascular structures. In [8], LUVS-Net was developed, a lightweight U-Net-based framework designed to reduce computational complexity while maintaining competitive segmentation performance. In [9], MSR U-Net was proposed, incorporating multiscale residual blocks and modified skip connections to improve the representation of vessels with different thicknesses and complex morphologies. In [10], a full-scale representation-guided network was introduced to enhance feature integration and improve the segmentation of fine vascular structures. Collectively, these developments indicate that architectural improvements remain an important research direction, particularly for addressing low-contrast vessels, morphological variability, and limited training data.

Despite these advances, segmentation accuracy obtained on individual benchmark datasets does not necessarily establish robust performance on images acquired under different conditions. Differences in imaging devices, resolution, illumination, contrast, and retinal vessel appearance can introduce distribution shifts that affect model generalization. To address this challenge, ref. [11] proposed UKnow-Net, which uses knowledge distillation from multiple dataset-specific teacher networks to improve segmentation across retinal image datasets. Such approaches demonstrate growing interest in improving robustness across heterogeneous imaging environments. However, methods that incorporate information from multiple datasets or use target-domain adaptation address a different experimental setting from source-only evaluation on an entirely unseen target dataset. Motivated by these considerations, the present study focuses on evaluating cross-dataset transfer rather than introducing a substantially modified segmentation architecture. Specifically, a U-Net model incorporating an ImageNet-pretrained ResNet34 encoder is trained using DRIVE images and subsequently evaluated on the independent CHASE_DB1 dataset without target-domain retraining or fine-tuning [12]. This experimental design provides a focused assessment of the model’s performance when transferred from its source dataset to a different retinal imaging dataset. By reporting both internal DRIVE validation and external CHASE_DB1 results, the study aims to characterize the limitations of source-trained retinal vessel segmentation under cross-dataset evaluation and highlight the importance of independent testing when assessing model generalization.

CNNs and U-Net variants have strong records for accuracy on benchmark datasets [13]; architectures based on the U-Net framework have emerged as predominant solutions due to their capacity to automatically learn hierarchical feature representations from training data [14,15]. Despite this, models in practical clinical settings often face performance degradation. Galdran et al. revealed that models struggle with cross-domain generalization and intricate vascular structures in real-world scenarios [16]. Unsupervised methodologies represent the genesis of automated vessel extraction, without reliance on manually annotated data. These approaches do not actually learn from data; instead, they operate on a set of fixed geometric rules derived from classical image processing theories. In [17], the authors presented a classic review on deep learning based retinal vessel segment mentation. In [18], a method is proposed that combines Gabor filters with fractional derivatives to improve vessel edges to separate vessels from the background. However, multilevel derivatives are highly sensitive to image noise and pathological artefacts, and the initial output typically requires additional steps such as morphological reconstruction to remove false positives in cluttered regions of the fundus [19,20]. Supervised machine learning methods represent a key shift in retinal vessel segmentation because they rely on annotated datasets. The models are trained on retinal images with corresponding ground truth annotations, which allows them to learn a discriminative mapping between image-derived features and vessel labels [21,22,23,24]. This enables the methods to capture more complex patterns, leading to improved segmentation accuracy. The classical supervised approach is categorized into two stages. In the first stage, handcrafted features are extracted from each pixel, including raw intensity values, colour channel information, gradient magnitude, and texture descriptors [5]. In the second stage, a statistical classifier is trained to assign each pixel to either the vessel or background class. The classifiers commonly used include k-nearest neighbors, support vector machines, decision trees, random forests, and boosting-based methods [25,26]. With the emergence of deep learning, retinal vessel segmentation was transformed through end-to-end learning of complex features. Convolutional neural networks (CNNs) do not require manual feature engineering as they automatically learn patterns from raw pixel data [27]. The standard CNN architecture was adapted from natural image analysis in early deep learning applications for vessel segmentation. U-Net has become the dominant framework for retinal vessel segmentation. Dense connections, which allow each layer to receive input from all previous layers, have been introduced to improve feature reuse and information flow [28]. Residual connections have been incorporated to improve gradient flow and training stability in deeper architectures [29]. Attention mechanisms have been introduced to guide the network toward relevant regions while suppressing background noise. In [30] the authors proposed an attention-based U-Net that improves segmentation performance by emphasizing salient vessel features. Rong et al. [18] also integrated spatial attention modules and self-calibrated convolutions to better capture vessel continuity and branching patterns. In [31], an improved U-Net model was proposed that incorporates multiscale context aggregation, resulting in improved detection of thin vessels. In [32], Vessel-Net was introduced, which uses multi-path supervision to incorporate features from varying depths of the network, further enhancing segmentation accuracy. Generative adversarial networks [33] have also been used to create synthetic retinal images and vessel maps to improve training data and prevent overfitting in low-data settings. Domain adaptation strategies have been explored to improve performance across datasets with different imaging conditions [34]. Most deep learning approaches rely heavily on supervised training and require large volumes of high-quality annotated vessel masks, which are costly, time-consuming to produce, and inconsistent. Semi-supervised learning, synthetic data generation using GANs, and self-labelling strategies have been introduced to reduce annotation demands, but these methods do not fully eliminate the dependence on labelled data and often introduce additional complexity into the training pipeline. The existing retinal vessel segmentation methods continue to face unresolved challenges related to thin vessel preservation, structural continuity, cross-dataset generalization, data dependency, and deployment efficiency. Addressing these limitations is essential for improving segmentation reliability in real-world clinical settings, as presented in this study.

## 3. Our Proposed Method

**Figure 1:**
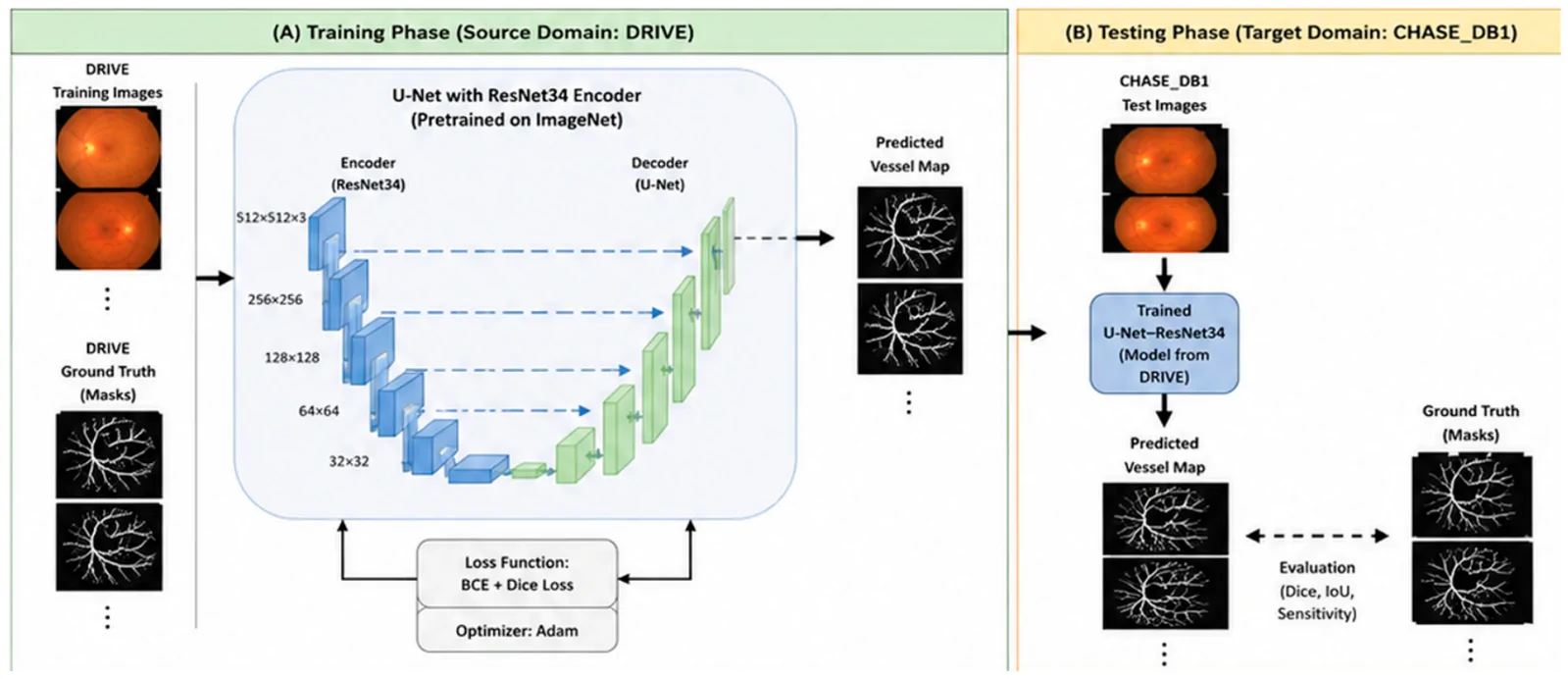
Overview of the experimental pipeline.

This study adopts a cross-dataset experimental framework to evaluate the robustness of deep learning-based retinal vessel segmentation. Rather than introducing a completely new architecture, the proposed approach uses a well-established U-Net segmentation network with an ImageNet-pretrained ResNet34 encoder and evaluates its ability to generalize from one retinal imaging dataset to another. The DRIVE dataset is used as the source domain for model training and internal validation, while CHASE_DB1 is used exclusively as an independent target-domain test set. The overall framework is illustrated in Figure 1 and detailed in Algorithm 1.
**Algorithm 1** Cross-Dataset Evaluation Pipeline. **Input:**    DRIVE training images I_D, DRIVE masks M_D, CHASE_DB1 test images I_C, CHASE_DB1 masks M_C **Output:**    Predicted vessel maps on I_C and performance metrics **1: Data Preparation** 1.1   Resize RGB fundus images to 256 × 256 pixels using bilinear interpolation. 1.2   Resize ground-truth vessel masks to 256 × 256 pixels using nearest-neighbor interpolation. 1.3   Normalize RGB image intensities to [0, 1] by dividing by 255. 1.4   Binarize vessel masks using a threshold of 0.5. **2: Model Initialization** 2.1   Load ImageNet-pretrained ResNet34 as encoder. 2.2   Construct U-Net with skip connections. **3: Training (on DRIVE)** 3.1   Split 20 DRIVE training images into 16 training and 4 validation images. 3.2   Apply spatial and photometric augmentation to training data only. 3.3   For each epoch do 3.4       Forward pass on I_D → predicted map P_D 3.5       Compute loss: L = BCE(P_D, M_D) + DiceLoss(P_D, M_D) 3.6       Update model weights using Adam optimizer. 3.7       Evaluate Dice coefficient on DRIVE validation images. 3.8   End For 3.9   Save model with best validation Dice score. **4: Inference (on CHASE_DB1)** 4.1   For each image I_C in CHASE_DB1 do 4.2       Predict vessel probability map P_C using trained model. 4.3       Binarize P_C using threshold 0.5. 4.4   End For **5: Evaluation** 5.1   Compute Dice, IoU, and sensitivity between binary P_C and M_C. 6: Return   Predicted vessel maps and performance metrics.

The proposed framework consists of two main phases: source-domain training and target-domain evaluation. In the first phase, retinal fundus images from DRIVE are used to train and validate the U-Net–ResNet34 model. Of the 20 available DRIVE training images, 16 are used for model training, and 4 are retained for validation. The validation subset is used to monitor model performance and reduce overfitting during training. In the second phase, the trained model is directly evaluated on CHASE_DB1 without additional retraining, fine-tuning, or domain adaptation. CHASE_DB1 therefore represents an unseen image domain containing differences in image resolution, patient population, contrast, and acquisition conditions. This train-on-DRIVE and test-on-CHASE_DB1 design enables direct assessment of cross-dataset generalization.

In this research, we have used a total of 20 training images. Of these, 16 are used for model training, and the remaining 4 are held out as a validation set to monitor overfitting during training. The CHASE_DB1 dataset was collected as part of a cardiovascular health study in school children in the United Kingdom [9]. It consists of 28 color fundus images with a resolution of 999 × 960 pixels, stored in JPEG format. The Table 1 show the key features of both datasets. The differences in resolution, image format, and acquisition conditions between DRIVE and CHASE_DB1 represent a domain shift to measure cross-dataset generalization. 

### 3.1. Preprocessing Pipeline

A uniform preprocessing pipeline is applied to all images from both datasets prior to training and evaluation. Applying the same pipeline to both datasets, specific preprocessing would artificially reduce the domain gap and inflate cross-dataset performance estimates. All colour fundus images are resized to 256 × 256 pixels to standardize input dimensions across both datasets and reduce memory requirements during training. Resizing is performed using bilinear interpolation. Pixel intensities are subsequently normalized to the range [0, 1] by dividing by 255, as expressed in Equation (1):

(1) $$\hat{x}=x/255$$

where x denotes the original pixel intensity value and $\hat{x}$ denotes the normalized value. This normalization ensures stable gradient computation during backpropagation and is consistent with standard practice in deep learning-based medical image analysis [1,9].

Ground truth vessel masks are loaded as grayscale images and hard-binarized using a threshold of 0.5, as expressed in Equation (2):

(2) $$\hat{m}=1\left[m>0.5\right]$$

where m is the normalized mask pixel value and $\hat{m}$ is the resulting binary label. This step is applied after resizing to correct for any interpolation artefacts introduced when scaling the binary mask. Nearest-neighbour interpolation is used during mask resizing to preserve sharp vessel boundaries and avoid the introduction of fractional values.

### 3.2. Data Augmentation

Due to the small size of the DRIVE training set (16 images), data augmentation is applied during training to reduce overfitting and improve the diversity of the training distribution. Random horizontal flips, random rotations, elastic deformations, colour jitter, and vertical flips are applied jointly to the image and its corresponding mask in a single operation, ensuring that spatial correspondence between input and label is maintained at all times. Augmentation is applied only to the training set; the validation and test sets are never augmented, in accordance with standard evaluation practice [1,12]. U-Net follows an encoder–decoder design with symmetric skip connections between corresponding layers of the encoder and decoder paths. The encoder progressively down-samples the input through convolution and pooling operations to extract hierarchical feature representations, while the decoder up-samples these features back to the original resolution.

Skip connections concatenate feature maps from the encoder directly to the corresponding decoder layers, preserving fine-grained spatial details such as thin vessel boundaries. The encoder of the U-Net is replaced with a ResNet34 backbone pretrained on the ImageNet dataset. ResNet34 is based on the residual learning framework proposed by He et al. [23].

The pretrained encoder encodes low-level visual features including edges, textures, and curvilinear structures from training on over one million natural images. When fine-tuned on retinal data, these representations provide a strong initialization that reduces the amount of labelled data required for effective learning [6,9]. The decoder weights are randomly initialized and trained from scratch on DRIVE data alongside the fine-tuned encoder. The output layer produces per-pixel probability maps in the range [0, 1], which are subsequently thresholded at 0.5 to produce binary vessel masks. The total parameter count of the model is approximately 21 million. The loss function, a combined Binary Cross-Entropy and Dice loss, is employed as the training objective, as described below. Binary Cross-Entropy (BCE) measures the per-pixel log-likelihood of the prediction given the ground truth label, as defined in Equation (3):

*L*_*BCE* = −(1/*N*) *Σ* [*y*_*i* · *log*(ŷ_*i*) + (1 − *y*_*i*) · *log*(1 − ŷ_*i*)] (3)

where N is the total number of pixels, y_i ∈ {0, 1} is the ground truth label for pixel i, and ŷ_i ∈ (0, 1) is the predicted probability. The Dice Similarity Coefficient (DSC) directly optimizes the overlap between the predicted and ground truth segmentation masks, as defined in Equations (4) and (5):

*DSC* = (2 · *Σ y*_*i* · *ŷ*_*i* + *ε*)/(*Σ y*_*i* + *Σ ŷ*_*i* + *ε*) (4)

*L*_*Dice* = 1 − *DSC* (5)

where ε is a small smoothing constant (1 × 10^−6^) that prevents numerical instability when both the prediction and ground truth are empty; the Dice is computed over the entire image rather than per pixel; it is inherently robust to class imbalance, as vessel and background pixels contribute equally to the overlap measure regardless of their relative frequency [1,7]. The final training objective is the sum of BCE and Dice losses, as expressed in Equation (6):

*L*_*total* = *L*_*BCE* + *L*_*Dice* (6)

BCE ensures per-pixel stability and correct probability adjustment, while Dice directly optimizes the segmentation overlap metric most relevant to clinical evaluation.

The Dice Coefficient, referred to as the F1 score in binary segmentation, measures the harmonic mean of precision and recall with respect to the vessel class, as defined in Equation (7):

Dice = 2TP/(2TP + FP + FN)(7)

where TP, FP, and FN denote the number of true positive, false positive, and false negative vessel pixels, respectively.

The false positives and false negatives are defined as in Equation (8):

IoU = TP/(TP + FP + FN)(8)

Sensitivity, referred to as recall or the true positive rate, measures the proportion of actual vessel pixels that are correctly detected, as defined in Equation (9):

*Sensitivity* = *TP*/(*TP* + *FN*) (9)

Sensitivity is of particular clinical relevance because a missed vessel (false negative) is more consequential in diabetic retinopathy screening than a false positive. This metric is the most sensitive to the detection of thin capillaries, which are disproportionately represented in the false negative count when a model fails to detect fine vascular structures [11,15,19]. Table 2 presents the qualitative monitoring of segmentation quality throughout the training process summa-rizes the complete training configuration.

Formally, the Grad-CAM saliency map C for a target output is defined as Equation (10):

*C* = *ReLU*(*Σ*_*k α*_*k* · *A*^*k*) (10)

where *A*^*k* denotes the k-th feature map of the target convolutional layer.

Grad-CAM is applied to both DRIVE and CHASE_DB1 samples to examine whether the model’s spatial attention changes when applied to an unseen domain. For example, a reduction in thin vessel regions in CHASE_DB1 images provides evidence of domain-specific feature dependence and helps to observe performance degradation on the cross-domain test set.

## 4. Simulation Results and Experiments

The experimental evaluation was conducted in two stages. In the first stage, a baseline U-Net v0.7.5 model was trained on the DRIVE dataset using a simple training pipeline without a validation split. A total of 20 DRIVE training images were used for model training with no held-out validation set. The model was compiled with the combined BCE and Dice loss and trained for the full 800 epochs without stopping early. The ResNet34 ImageNet-pretrained weights were loaded prior to training.

**Figure 2:**
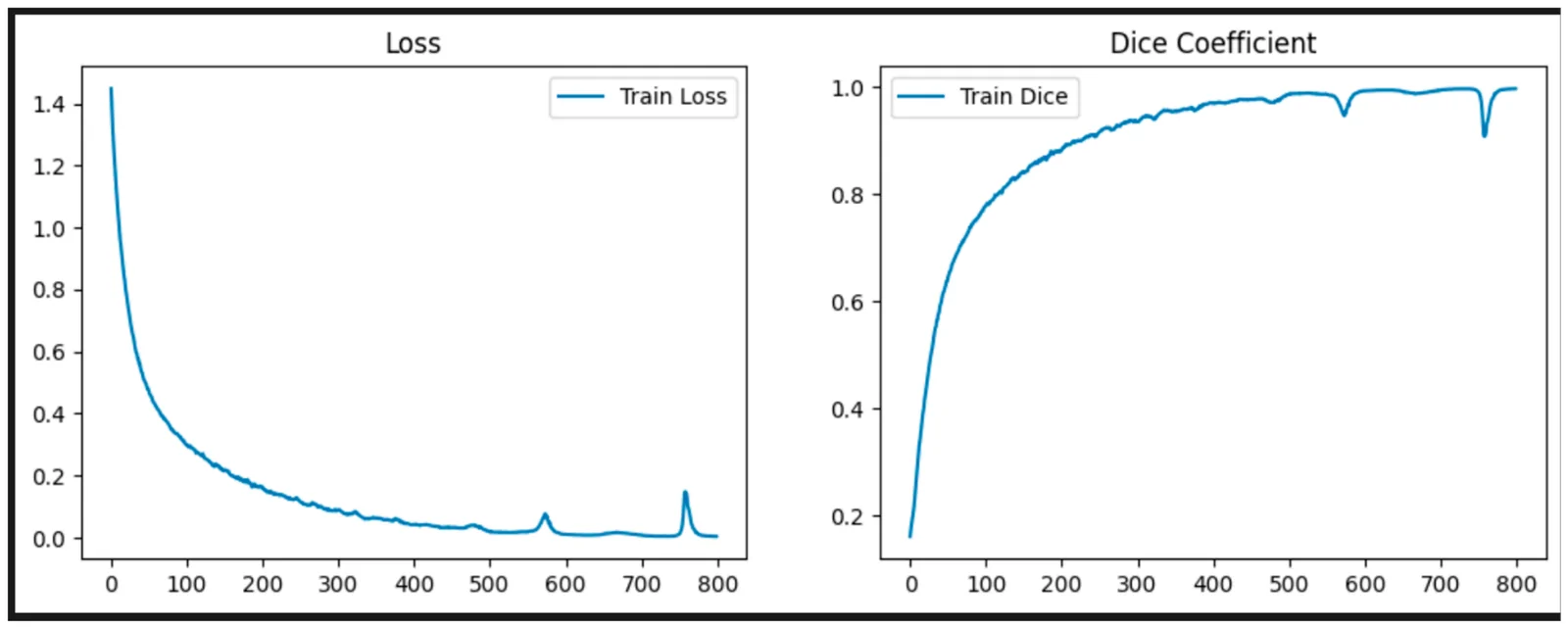
Training curves showing loss (**left**) and Dice coefficient (**right**) over 800 epochs.

**Figure 3:**
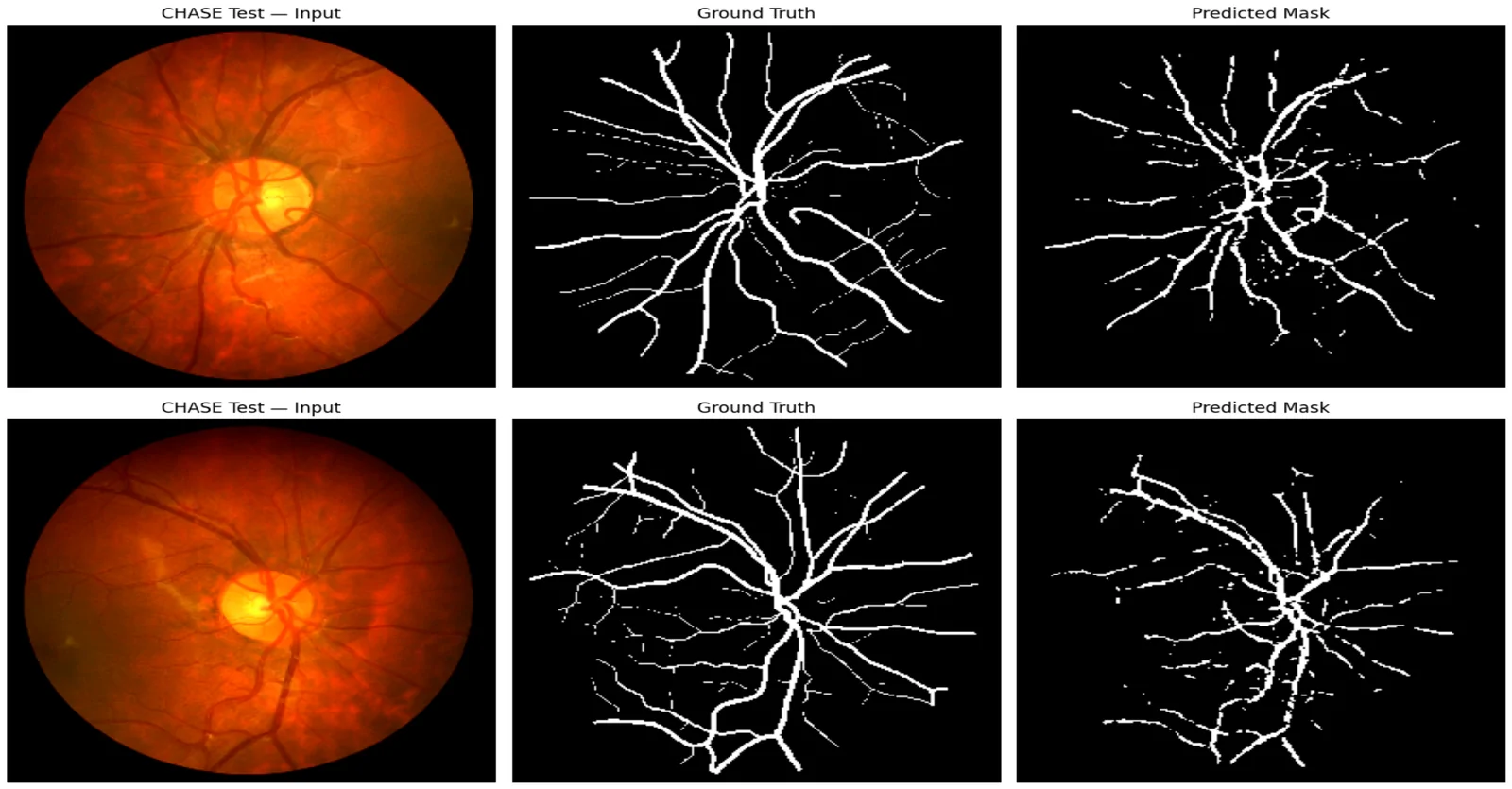
Qualitative segmentation results of the proposed U-Net–ResNet34 model on the CHASE_DB1 external test dataset. Each row presents a representative retinal fundus image (**left**), the corresponding ground-truth vessel mask (**middle**), and the predicted segmentation mask (**right**).

**Figure 4:**
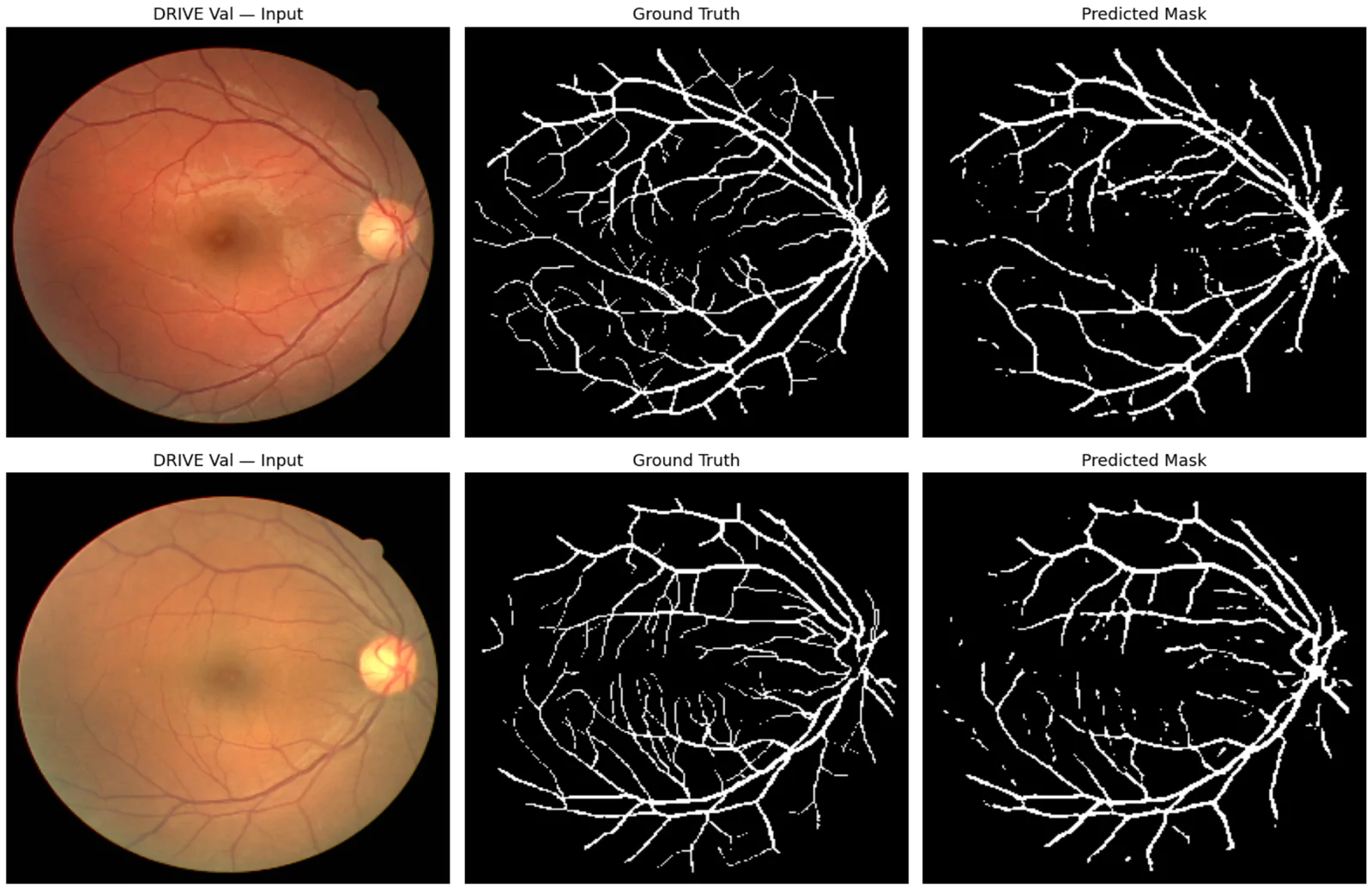
Qualitative segmentation results of the proposed U-Net–ResNet34 model on the DRIVE validation dataset. Each row presents a representative retinal fundus image (**left**), the corresponding ground-truth vessel mask (**middle**), and the predicted segmentation mask (**right**).

**Figure 5:**
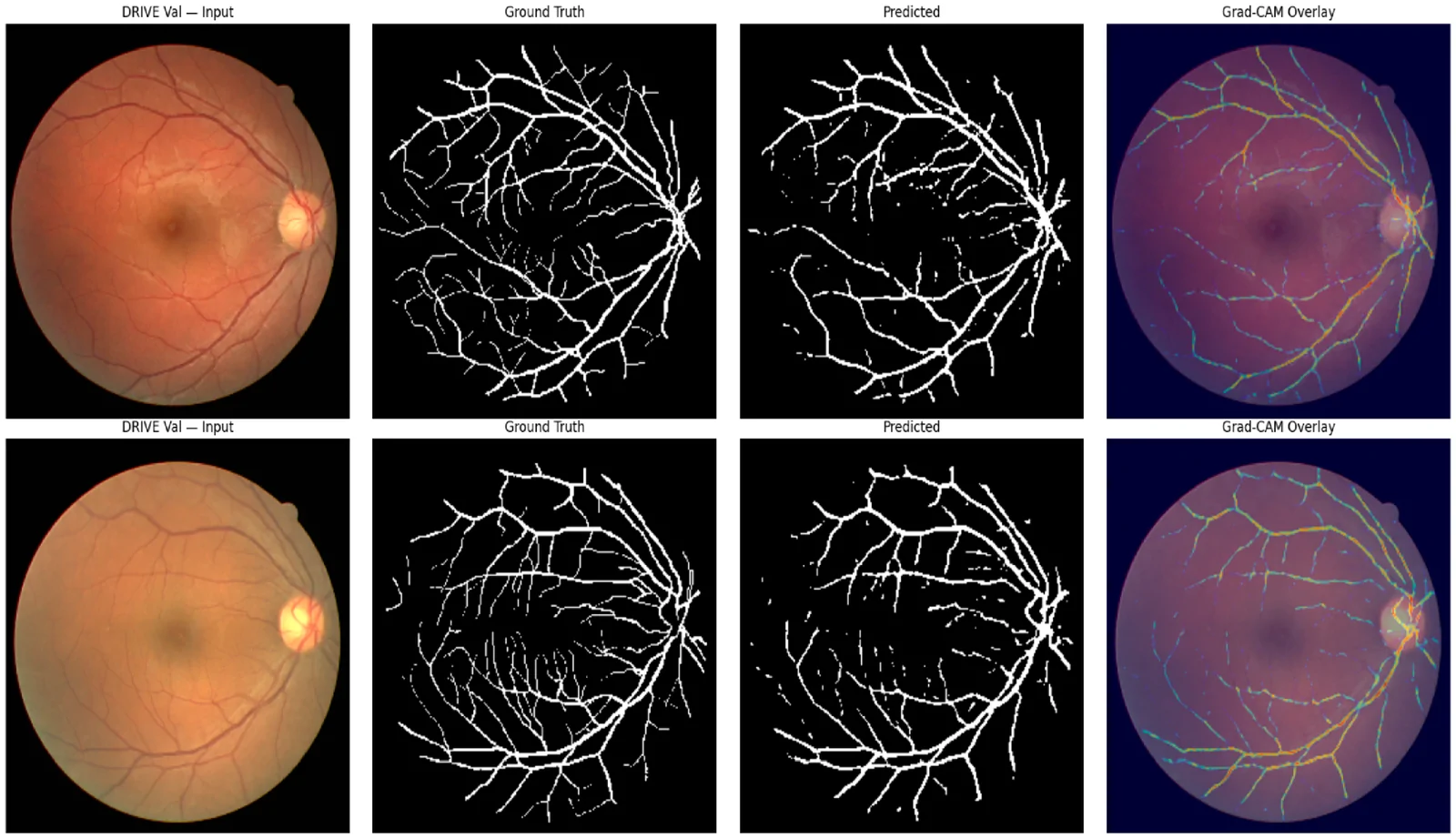
Grad-CAM overlay (Drive) image segmentation.

**Figure 6:**
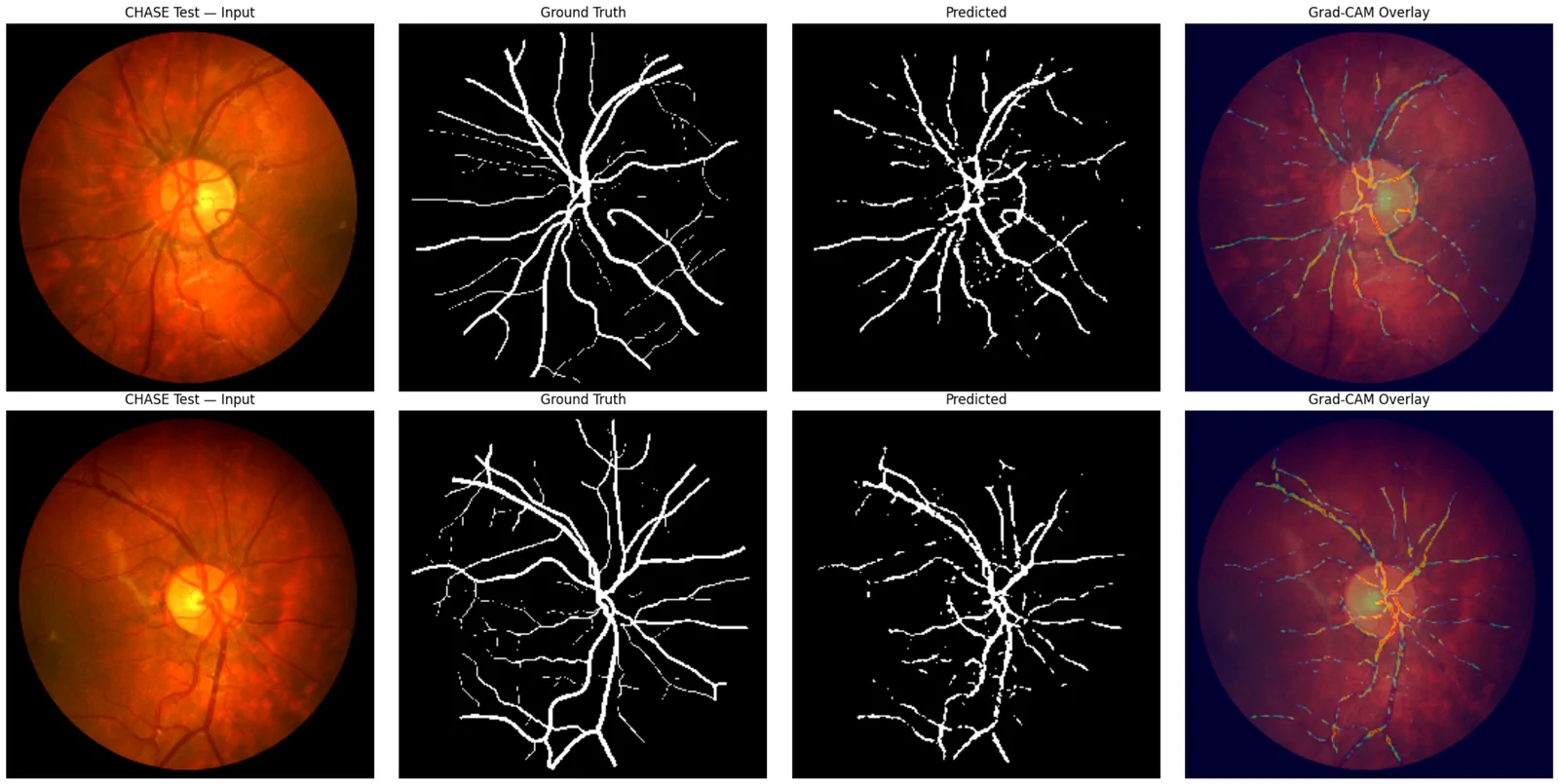
Grad-CAM overlay (Chase_DB1) image segmentation.

Figure 2 presents the training loss and Dice coefficient over 800 epochs. The loss curve shows a rapid decrease from approximately 1.44 at epoch 1 to below 0.1 by epoch 300, after which it continues to decline gradually toward near zero by epoch 800. The Dice coefficient curve rises steeply from approximately 0.17 at epoch 1 to 0.90 by epoch 300, and continues to increase to approximately 0.997 by epoch 800. Since no validation set is used, it is not possible to determine from this curve whether the model overfitted to the training data—the prediction samples are from both the DRIVE validation set and the CHASE_DB1 test set. Figure 3 and Figure 4 illustrate representative qualitative segmentation results obtained from the CHASE_DB1 external test dataset and the DRIVE validation dataset, respectively. Each figure presents the original retinal fundus images, their corresponding ground-truth vessel annotations, and the segmentation masks predicted by the proposed U-Net–ResNet34 model. Visual inspection indicates that the model identifies several major retinal vessels in both datasets. However, differences between the predicted and reference masks are apparent, particularly in thin vascular branches and low-contrast regions. The qualitative results complement the quantitative evaluation and illustrate the challenges associated with transferring a model trained on DRIVE to the independent CHASE_DB1 dataset. On the DRIVE validation samples, the predicted masks closely follow the ground truth for major vessel branches. Some thin peripheral capillaries are missed or fragmented in the predictions, particularly in the second sample where the ground truth shows a dense network of fine vessels in the lower half of the image as shown in Table 3. Overall, the spatial structure of the vasculature is well preserved in the DRIVE predictions. These assessments are consistent with the quantitative sensitivity drop from 0.72 to 0.55, and the thin vessel recall is disproportionately affected by the domain shift from DRIVE to CHASE_DB1. Grad-CAM overlays for two DRIVE validation samples and two CHASE_DB1 test samples show the spatial attention of the model alongside the ground truth and predicted mask. For the DRIVE validation samples, the Grad-CAM heatmaps show concentrated activation along the major vessel pathways, with the brightest activations (yellow) corresponding to thick vessels near the optic disc and progressively cooler (green and blue) along thinner vessels toward the periphery. This pattern indicates that the model attends most strongly to the high-contrast vessel structures shown in Figure 4, Figure 5 and Figure 6. On the CHASE_DB1 test samples, the Grad-CAM patterns are visually similar in overall structure and remain centered on major vessels.

To further assess the generalization capability and reliability of the proposed U-Net–ResNet34 segmentation model, an independent within-domain evaluation using the official DRIVE test set is considered. The DRIVE dataset consists of 40 retinal fundus images, divided into 20 training images and 20 independent test images. In the present study, 16 images from the official training partition were used for model optimization, while the remaining four images were reserved for internal validation and model selection based on the Dice similarity coefficient. Although the internal validation results provide an initial assessment of segmentation performance, the limited number of validation images may introduce substantial sampling variability and may not adequately represent the diversity of retinal vascular structures within the dataset. Therefore, evaluation on the official DRIVE test partition is important for obtaining a more reliable estimate of within-domain segmentation performance using images that were not involved in model training or checkpoint selection. For this evaluation, the model checkpoint selected using the DRIVE validation subset should be applied directly to all 20 official DRIVE test images without additional training, fine-tuning, or hyperparameter optimization. The same preprocessing and inference procedures described in Section 3 should be maintained, including resizing fundus images to 256 × 256 pixels, pixel-intensity normalization, and binary segmentation using a probability threshold of 0.5. Segmentation performance should be quantified using the Dice similarity coefficient, intersection over union (IoU), and sensitivity. The independent DRIVE test results would provide an important reference for interpreting the external evaluation on CHASE_DB1. Specifically, comparing the model’s performance on the official DRIVE test set and CHASE_DB1 would help distinguish segmentation limitations observed within the source imaging domain from additional performance degradation associated with differences in acquisition characteristics, image resolution, contrast, and vascular appearance across datasets. Furthermore, reporting image-level performance variability, including standard deviations or confidence intervals, would improve the statistical interpretation of the results. Such evaluation is particularly important given the relatively small number of images available for model development. Overall, independent within-domain testing would strengthen the experimental assessment and provide a more robust basis for interpreting the cross-dataset generalization capability of the proposed segmentation framework. Table 4 shows a comparison of segmentation performance.

Figure 5 presents the Grad-CAM overlay (Drive) image segmentation and Figure 6 shows the Grad-CAM overlay (Chase_DB1) image segmentation.

Along thin vessels is noticeably reduced compared to the DRIVE samples. The learned vessel features that are specific to the DRIVE domain do not transfer as effectively to the higher-resolution, differently contrasted CHASE_DB1 images. The reduced activation in peripheral vessel regions on CHASE_DB1 is directly reflected in the increased false negative rate observed in the quantitative results. The cross-domain Dice of 0.6269 on CHASE_DB1 represents the model’s genuine generalization capability when evaluated on unseen data from a different imaging domain. The evaluation framework includes a validation split, early stopping, hard metric computation, and cross-domain testing on CHASE_DB1. The improved pipeline produced a validation Dice of 0.7263 on DRIVE and a cross-domain Dice of 0.6269 on CHASE_DB1, with a sensitivity drop from 0.7201 to 0.5502 between the two datasets. Galdran et al. [4] reported that benchmark scores in retinal vessel segmentation are often inflated because models are evaluated on the same data they are trained on, or because soft metrics are used instead of hard ones. There was a drop in performance when the model was tested on CHASE_DB1. The Dice dropped from 0.7263 on DRIVE validation to 0.6269 on CHASE_DB1. The IoU dropped from 0.5703 to 0.4573, and sensitivity dropped from 0.7201 to 0.5502. When images are 256 by 256 pixels, a thin capillary might only be two or three pixels wide. At that scale, it becomes very easy for pooling operations to lose the vessel entirely [18].

Table 5 shows training on the full 20-image DRIVE training set and testing on a separate 20-image test set. The small dataset size for a deep learning model with roughly 24 million parameters means the model clearly overfits, as shown by the training–validation Dice gap of around 0.22 points. A cross-validation approach over multiple splits would give a more stable and reliable estimate of performance. The augmentation pipeline likely improves both in-domain validation performance and cross-domain generalization. CLAHE preprocessing in particular has been shown to improve vessel contrast in fundus images and increase sensitivity to thin vessels [11]. Adding elastic deformations and random rotations to the pipeline exposed the model to a wider variety of vessels.

The consequences of this study assist as a reference point for upcoming work on large datasets with domain adaptation and thin vessel detection in retinal vessel segmentation.

**Figure 7:**
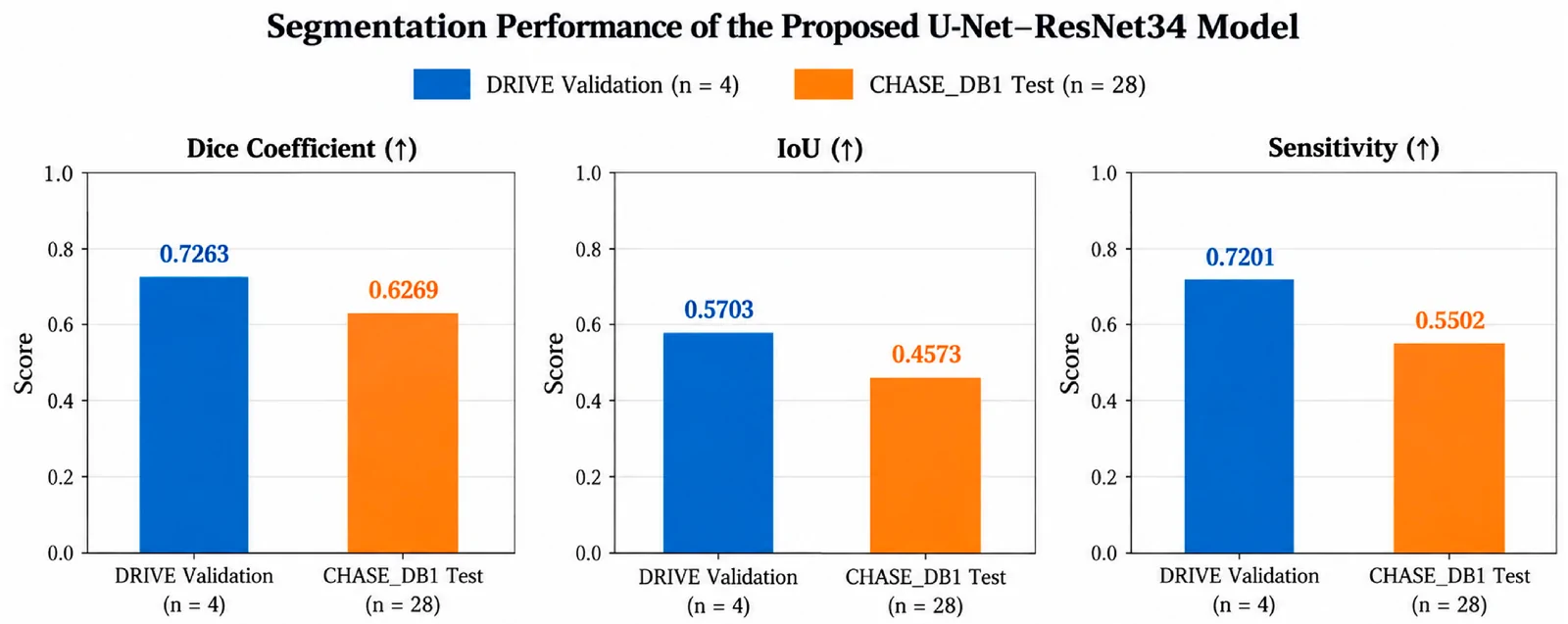
Comparison with state-of-the-art models.

Figure 7 illustrates the quantitative segmentation performance of the proposed U-Net–ResNet34 model on the DRIVE validation subset and the independent CHASE_DB1 external test dataset using three evaluation metrics: Dice similarity coefficient, intersection over union (IoU), and sensitivity. On the DRIVE validation subset (n = 4), the proposed model achieved a Dice coefficient of 0.7263, an IoU of 0.5703, and a sensitivity of 0.7201, demonstrating its ability to identify retinal vascular structures within the source imaging domain. When evaluated on CHASE_DB1 (n = 28) without additional training, fine-tuning, or target-domain adaptation, the model achieved a Dice coefficient of 0.6269, an IoU of 0.4573, and a sensitivity of 0.5502. Compared with the DRIVE validation results, the observed performance decreased by 0.0994 in Dice, 0.1130 in IoU, and 0.1699 in sensitivity. The larger reduction in sensitivity suggests that the model identified a smaller proportion of annotated vessel pixels under the external evaluation condition, potentially reflecting difficulties in detecting thin, low-contrast, or structurally complex retinal vessels. These differences may be associated with variations in image acquisition characteristics, illumination, resolution, and vessel appearance between the datasets. However, because the DRIVE results were obtained from only four validation images rather than the official independent DRIVE test set, the observed differences should be interpreted descriptively rather than as a definitive estimate of the effect of dataset shift. Overall, the findings provide preliminary evidence that the proposed model can transfer to an independent retinal image dataset without target-domain retraining, while highlighting limitations in its external segmentation performance. The results emphasize the importance of evaluating retinal vessel segmentation models across independent datasets and reporting clearly defined evaluation protocols and measures of statistical variability to support reliable conclusions about generalization.

### Statistical and Comparative Analysis

To further characterize the performance of the proposed U-Net–ResNet34 model, an additional descriptive analysis was conducted using the reported Dice similarity coefficient, intersection over union (IoU), and sensitivity values obtained from the DRIVE validation subset and CHASE_DB1 external evaluation dataset. The DRIVE validation results were 0.7263 for Dice, 0.5703 for IoU, and 0.7201 for sensitivity. Under external evaluation on CHASE_DB1, the corresponding values decreased to 0.6269, 0.4573, and 0.5502, respectively. These differences represent absolute reductions of 0.0994 in Dice, 0.1130 in IoU, and 0.1699 in sensitivity, corresponding to relative reductions of 13.69%, 19.81%, and 23.59%. Among the three metrics, sensitivity exhibited the greatest relative reduction. This observation indicates that a smaller proportion of annotated vessel pixels was correctly identified during external evaluation. Such a reduction may be associated with difficulties in detecting thin or low-contrast vessels under differences in image acquisition characteristics and retinal appearance. However, the observed changes should be interpreted descriptively. The DRIVE results were obtained from only four validation images, while CHASE_DB1 represented a separate external evaluation dataset. Consequently, the reported differences do not constitute a controlled paired comparison, and no conclusions regarding statistical significance can be drawn from the aggregate scores alone. Image-level metric distributions and additional independent evaluations would be required to quantify uncertainty more comprehensively.

## 5. Discussions on Influence of Image Resolution and Quality on Cross-Dataset Generalization

Image resolution and acquisition quality are important considerations in retinal vessel segmentation because the vascular network contains thin, low-contrast structures that may be difficult to distinguish from the surrounding retinal background. In the present study, both DRIVE and CHASE_DB1 images were resized to a common resolution of 256 × 256 pixels and processed using the same normalization procedure to maintain consistency across the source and target datasets. Although this approach supports standardized evaluation, spatial downsampling may reduce the visibility of small vessels and fine vascular branches. Similarly, image blur, variations in illumination, and reduced contrast may affect vessel boundary definition and the identification of low-intensity vascular structures. The observed decrease in Dice coefficient, IoU, and sensitivity between DRIVE validation and CHASE_DB1 external testing is consistent with the possibility that differences in image characteristics influence segmentation performance. However, these differences cannot be attributed specifically to resolution, blur, or image quality because the effects of these factors were not isolated experimentally. Furthermore, the DRIVE results were obtained from a small validation subset, which limits the strength of direct conclusions about the magnitude of cross-dataset performance degradation. Future work should therefore incorporate controlled sensitivity experiments involving alternative image resolutions, different Gaussian blur levels, and systematic brightness and contrast perturbations. Such experiments would help quantify the contribution of individual image-quality factors to segmentation performance and provide a more comprehensive assessment of model robustness under heterogeneous retinal imaging conditions.

### Limitations and Future Directions

An important limitation of the present study is the relatively small number of annotated retinal images used for model development. The U-Net–ResNet34 framework was trained on 16 DRIVE images, while only four images were used for internal validation. Although ImageNet-pretrained encoder weights were employed to support feature extraction under limited-data conditions, the restricted training sample may not adequately represent the full diversity of retinal vessel morphology, image contrast, illumination, and acquisition characteristics encountered in clinical practice.

The small validation subset also limits the precision and stability of internal performance estimates. In particular, the reported validation metrics may be sensitive to the selection of individual images, and the limited validation data may affect the reliability of model selection through early stopping. Consequently, the internal DRIVE validation results should not be interpreted as a comprehensive estimate of performance on unseen source-domain images. The use of a limited source dataset was motivated by the study’s specific objective of examining source-to-target transfer under a clearly defined benchmark protocol. Rather than combining multiple training datasets or incorporating target-domain examples, the model was trained exclusively using DRIVE and subsequently evaluated on CHASE_DB1 without target-domain adaptation. This design enabled a focused examination of model behavior under the selected cross-dataset evaluation setting. Nevertheless, the deliberate restriction of source-domain training data may have constrained the range of vascular and imaging features learned by the network. Although the external CHASE_DB1 evaluation provides useful evidence of model performance on an independent dataset, it does not establish robustness across broader clinical populations, acquisition devices, or retinal disease conditions. The observed reduction in external segmentation performance, particularly in sensitivity, further indicates the need for caution when interpreting the generalizability of the proposed framework.

Future investigations should therefore incorporate larger and more diverse annotated training datasets, additional independent external evaluation datasets, and repeated experiments using different training and validation partitions. Such extensions would help determine whether the observed performance differences persist across alternative samples and would provide stronger evidence regarding the reliability and generalizability of the proposed model.

## 6. Conclusions

This study evaluated the cross-dataset generalization of a deep-learning model for retinal vessel segmentation using a U-Net architecture with an ImageNet-pretrained ResNet34 encoder. The model achieved a Dice coefficient of 0.7263, IoU of 0.5703, and sensitivity of 0.7201 on the held-out DRIVE validation images. When applied without retraining or domain adaptation to the independent CHASE_DB1 dataset, performance decreased to a Dice coefficient of 0.6269, IoU of 0.4573, and sensitivity of 0.5502. The particularly large absolute reduction in sensitivity of approximately 0.17 indicates that substantially more reference vessel pixels were missed during cross-dataset evaluation. Qualitative assessment of the predicted vessel maps showed that major vascular branches were generally preserved, whereas smaller, low-contrast, and peripheral vessels appeared more susceptible to fragmentation and omission, particularly in CHASE_DB1 images. Because vessel-width-specific recall was not quantified, these observations should be interpreted as qualitative evidence of fine-vessel segmentation difficulty rather than as a direct measurement of thin-vessel performance. The findings demonstrate that transfer learning with an ImageNet-pretrained encoder does not eliminate the effects of dataset shift. Differences in image resolution, illumination, contrast, acquisition characteristics, and population composition may contribute to reduced performance when a model is applied outside its development domain. Consequently, performance obtained exclusively from images originating from the development dataset may provide an incomplete estimate of model robustness. Overall, this study highlights the importance of cross-dataset external evaluation when assessing deep-learning systems for retinal vessel segmentation. Robustness across heterogeneous imaging conditions should be considered alongside improvements in conventional benchmark performance when developing retinal vascular analysis systems. Future research should investigate higher-resolution processing, vessel-width-specific evaluation, larger and more diverse multicentre datasets, stronger augmentation strategies, multi-domain training, and domain-adaptation or domain-generalization approaches to improve the transferability of automated retinal vessel segmentation.

## Figures and Tables

**Table 1:** The key characteristics of both datasets.

Property	DRIVE	CHASE_DB1
Number of images used	20 (16 train/4 val)	28 (test only)
Image resolution	565 × 584 px	999 × 960 px
Image format	TIFF	JPEG
Mask format	GIF (binary)	PNG (binary)
Annotators per image	1	2 (1stHO used)
Field of view	45°	30°
Role in study	Training + Validation	Cross-domain Test

**Table 2:** Qualitative monitoring of segmentation.

Hyperparameter	Value
Optimiser	Adam
Learning rate	1 × 10^−4^
Batch size	2
Maximum epochs	800
Early stopping patience	100 epochs
Early stopping monitor	Validation Dice coefficient
Image size	256 × 256 pixels
Loss function	BCE + Dice (combined)
Output activation	Sigmoid
Framework	TensorFlow 2.x/Keras
Hardware	Google Colab GPU (T4)

**Table 3:** Final training metrics at epoch 800.

Metric	Value (Epoch 800)
Loss (BCE + Dice)	0.0043
Dice Coefficient (soft)	0.9970
IoU (soft)	0.9971
Sensitivity (soft)	0.9972

**Table 4:** Segmentation performance of the proposed U-Net–ResNet34 model under internal and external evaluation.

Evaluation Dataset	Number of Images	Dice (Mean ± SD)	IoU (Mean ± SD)	Sensitivity (Mean ± SD)
DRIVE validation	4	0.7263 ± TBD	0.5703 ± TBD	0.7201 ± TBD
CHASE_DB1 external test	28	0.6269 ± TBD	0.4573 ± TBD	0.5502 ± TBD

**Table 5:** Contextual comparisons of DRIVE and CHASE_DB1 Dice scores.

Study	Method	DRIVE Dice	CHASE Dice
Sun et al. [12]	Improved U-Net	~0.82	N/A (not applicable)
Li et al. [14]	Dense-U-Net	~0.81	N/A (not applicable)
Radha et al. [6]	Attention U-Net	~0.82	N/A (not applicable)
Galdran et al. [4]	Minimalistic W-Net	~0.82	Cross-dataset drop reported
Yue et al. [20]	Teacher–student (with DA)	~0.81	~0.79
Proposed	U-Net + ResNet34 (no DA)	0.7263	0.6269

## Data Availability

A public dataset is available at: https://www.kaggle.com/datasets/andrewmvd/drive-digital-retinal-images-for-vessel-extraction (accessed on 1 September 2026).

## References

[1] Chen C., Chuah J.H., Ali R., Wang Y. (2021). Retinal vessel segmentation using deep learning: A review. IEEE Access.

[2] Soomro T.A., Afifi A.J., Zheng L., Soomro S., Gao J., Hellwich O., Paul M. (2019). Deep learning models for retinal blood vessels segmentation: A review. IEEE Access.

[3] Chetoui M., Akhloufi M.A. (2025). A Novel Ensemble Meta-Model for Enhanced Retinal Blood Vessel Segmentation Using Deep Learning Architectures. Biomedicines.

[4] Galdran A., Anjos A., Dolz J., Chakor H., Lombaert H., Ben Ayed I. (2022). State-of-the-art retinal vessel segmentation with minimalistic models. Sci. Rep..

[5] Memari N., Ramli A.R., Saripan M.I.B., Mashohor S., Ismail R. (2019). Retinal blood vessel segmentation by using matched filtering and fuzzy C-means clustering with integrated level set method for diabetic retinopathy assessment. J. Med. Biol. Eng..

[6] Radha K., Yepuganti K., Saritha S., Kamireddy C., Bavirisetti D.P. (2023). Unfolded deep kernel estimation-attention U-Net-based retinal image segmentation. Sci. Rep..

[7] Radha K., Karuna Y. (2025). Latent space autoencoder generative adversarial model for retinal image synthesis and vessel segmentation. BMC Med. Imaging.

[8] Aguirre-Ramos H., Avina-Cervantes J.G., Cruz-Aceves I., Ruiz-Pinales J., Ledesma S. (2018). Blood vessel segmentation in retinal fundus images using Gabor filters, fractional derivatives, and Expectation Maximization. Appl. Math. Comput..

[9] Qin Q., Chen Y. (2024). A review of retinal vessel segmentation for fundus image analysis. Eng. Appl. Artif. Intell..

[10] Zhao J., Feng Q. (2021). Deep att-ResGAN: A retinal vessel segmentation network for color fundus images. Trait. Signal.

[11] Sule O., Viriri S. (2023). Contrast enhancement of RGB retinal fundus images for improved segmentation of blood vessels using convolutional neural networks. J. Digit. Imaging.

[12] Sun K., Chen Y., Chao Y., Geng J., Chen Y. (2023). A retinal vessel segmentation method based improved U-Net model. Biomed. Signal Process. Control.

[13] Laibacher T., Weyde T., Jalali S. M2u-net: Effective and efficient retinal vessel segmentation for real-world applications. Proceedings of the IEEE/CVF Conference on Computer Vision and Pattern Recognition Workshops.

[14] Li Z., Jia M., Yang X., Xu M. (2021). Blood vessel segmentation of retinal image based on dense-U-Net network. Micromachines.

[15] Nayagam R.D., Rahini A.A., Fathima A.F., Gomathi K.L. (2021). An Enhanced Retinal vessel segmentation using Deep Convolution Neural Network. J. Phys. Conf. Ser..

[16] Melinscak M., Prentasic P., Loncaric S. Retinal Vessel Segmentation using Deep Neural Networks. Proceedings of the VISAPP 2015—International Conference on Computer Vision Theory and Applications.

[17] Khandouzi A., Ariafar A., Mashayekhpour Z., Pazira M., Baleghi Y. (2022). Retinal vessel segmentation, a review of classic and deep methods. Ann. Biomed. Eng..

[18] Rong Y., Xiong Y., Li C., Chen Y., Wei P., Wei C., Fan Z. (2023). Segmentation of retinal vessels in fundus images based on U-Net with self-calibrated convolutions and spatial attention modules. Med. Biol. Eng. Comput..

[19] Liu Z., Sunar M.S., Tan T.S., Hitam W.H.W. (2025). Deep learning for retinal vessel segmentation: A systematic review of techniques and applications. Med. Biol. Eng. Comput..

[20] Yue K., Zhan L., Wang Z. (2025). Unsupervised domain adaptation teacher–student network for retinal vessel segmentation via full-resolution refined model. Sci. Rep..

[21] Wu Y., Xia Y., Song Y., Zhang D., Liu D., Zhang C., Cai W. (2019). Vessel-Net: Retinal vessel segmentation under multi-path supervision. International Conference on Medical Image Computing and Computer-Assisted Intervention.

[22] Ronneberger O., Fischer P., Brox T. (2015). U-Net: Convolutional networks for biomedical image segmentation. Medical Image Computing and Computer-Assisted Intervention (MICCAI).

[23] He K., Zhang X., Ren S., Sun J. Deep residual learning for image recognition. Proceedings of the IEEE Conference on Computer Vision and Pattern Recognition (CVPR).

[24] Abbasi R., Xu L., Wang Z., Chughtai G.R., Amin F., Luo B. Dynamic weighted histogram equalization for contrast enhancement using for Cancer Progression Detection in medical imaging. Proceedings of the 2018 International Conference on Signal Processing and Machine Learning.

[25] Staal J., Abramoff M.D., Niemeijer M., Viergever M.A., van Ginneken B. (2004). Ridge-based vessel segmentation in color images of the retina. IEEE Trans. Med. Imaging.

[26] Abbasi R., Amin F., Alabrah A., Choi G.S., Khan S., Bin Heyat M.B., Iqbal M.S., Chen H. (2025). Diabetic retinopathy detection using adaptive deep convolutional neural networks on fundus images. Sci. Rep..

[27] Abbasi M.J., Abbasi R., Wu S., Heyat M.B.B., Xianfeng D., Jia H., Zheng A. (2025). Machine learning modeling and analysis of prognostic hub genes in cervical adenocarcinoma: A multi target therapy for enhancement in immunosurveillance. Discov. Oncol..

[28] Selvaraju R.R., Cogswell M., Das A., Vedantam R., Parikh D., Batra D. Grad-CAM: Visual explanations from deep networks via gradient-based localization. Proceedings of the IEEE International Conference on Computer Vision (ICCV).

[29] Abbasi R., Chen J., Al-Otaibi Y., Rehman A., Abbas A., Cui W. (2021). RDH-based dynamic weighted histogram equalization using for secure transmission and cancer prediction. Multimed. Syst..

[30] Jun W., Huiqin C., Abbasi R., Iqbal M.S., Heyat M.B.B. (2025). Transforming physical healthcare training through integration of machine learning and advanced artificial intelligent methods. J. Hum. Sport Exerc..

[31] Ansari M.M., Kumar S., Chola C., Heyat B.B., Akhtar F., Hayat M.A.B., Sayeed E., Parveen S., Abbasi R., Pomary D. (2025). A Novel Machine and Deep Learning–Based Ensemble Techniques for Automatic Lung Cancer Detection. BioMed Res. Int..

[32] Abbasi R., Al-Otaibi Y.D., Yuan G. (2024). Efficient lossless secure communication for smart cities healthcare-based cyborg robots. Hum.-Centric Comput. Inf. Sci..

[33] Jadhav A., Awale R.N., Ingle R.R. (2026). ACNN-DR: Adaptive convolutional neural network for diabetic retinopathy using bias correction and continual learning. Biomed. Signal Process. Control.

[34] Hailu A.A., Desta E.A., Mulugeta A., Adugna F.B., Salau A.O., Almaw L.A., Fentahun M.B. (2026). Early retinal disease detection from fundus images using deep neural networks. Sci. Rep..

